# What Happens When the Digested Screw Does Not Come Out of Ileum?

**DOI:** 10.7759/cureus.20169

**Published:** 2021-12-04

**Authors:** Avleen Kaur, Kaveh Zivari, Neha Sharma

**Affiliations:** 1 Internal Medicine, Maimonides Medical Center, Brooklyn, USA; 2 Gastroenterology, Maimonides Medical Center, Brooklyn, USA

**Keywords:** screw, colonoscopic removal, asge, impaction in ileocecal, sharp object

## Abstract

Adults with foreign body ingestion are mainly secondary to psychiatric disorders, alcoholic intoxication, and secondary gains. Conservative management without any intervention is successful in 80% of the ingested foreign bodies. Risk factors for complication include sharp objects, objects larger than 6 mm, recurrent ingestion, and previous gastrointestinal tract surgeries. Sharp objects specifically account for 35% perforation rates and impactions, most commonly at the ileocecal valve. There is limited evidence on the role of colonoscopy after the distal migration of foreign bodies into the ileum and colon.

In our case report, we present a case of a 53-year-old-male with a history of recurrent foreign body ingestion secondary to a multitude of psychiatric disorders. It describes multiple foreign body ingestions, leading to failure of a screw at the ileocecal valve at day 5 of ingestion, despite conservative management with serial bowel preparations and abdominal radiographs. There is limited evidence on the management of foreign bodies after distal migration to the ligament of Trietz. Existing literature and guidelines suggest surgically managing the sharp foreign bodies after the failure of conservative management for three to five days.

In the case report, we have attempted to emphasize the noninvasive, colonoscopic approach as initial management in removing impacted foreign bodies.

This abstract has been presented and accepted at the American college of gastroenterology meeting held from October 22, 2021, to October 27, 2021, in Las Vegas as a poster.

## Introduction

The common scenarios where adults ingest a true foreign body (i.e., nonfood object) are psychiatric disorders, alcohol intoxication, and incarcerated individuals seeking secondary gains [1]. The preendoscopic series concluded that 80% or more foreign bodies pass without intervention, with mortality rates under 0.1% [2]. However, endoscopic interventions are higher (62-76%) in patients with an intentional ingestion of foreign bodies than unintentional [1,3]. Patients with recurrent foreign body ingestion and previous gastrointestinal tract surgeries are at higher risk of impactions, perforation, and obstruction [4,5]. Sharp objects raise concern for perforation rates to 35%, commonly at the ileocecal valve and less commonly at appendiceal lumen, cecal-ascending colon junction, colonic flexures, and rectosigmoid junction. Objects larger than 6 cm impose a risk for small bowel obstruction [2,6]. The American Society for Gastrointestinal Endoscopy (ASGE) has well-established guidelines stating emergent endoscopic management of sharp foreign bodies in the esophagus and urgent removal of objects in the stomach and duodenum. However, there is limited evidence on the role of colonoscopy after the distal migration of foreign bodies into the colon [7].

We present a middle-aged male with successful colonoscopic removal of an impacted foreign body after conservative management for two weeks.

## Case presentation

**Figure 1:**
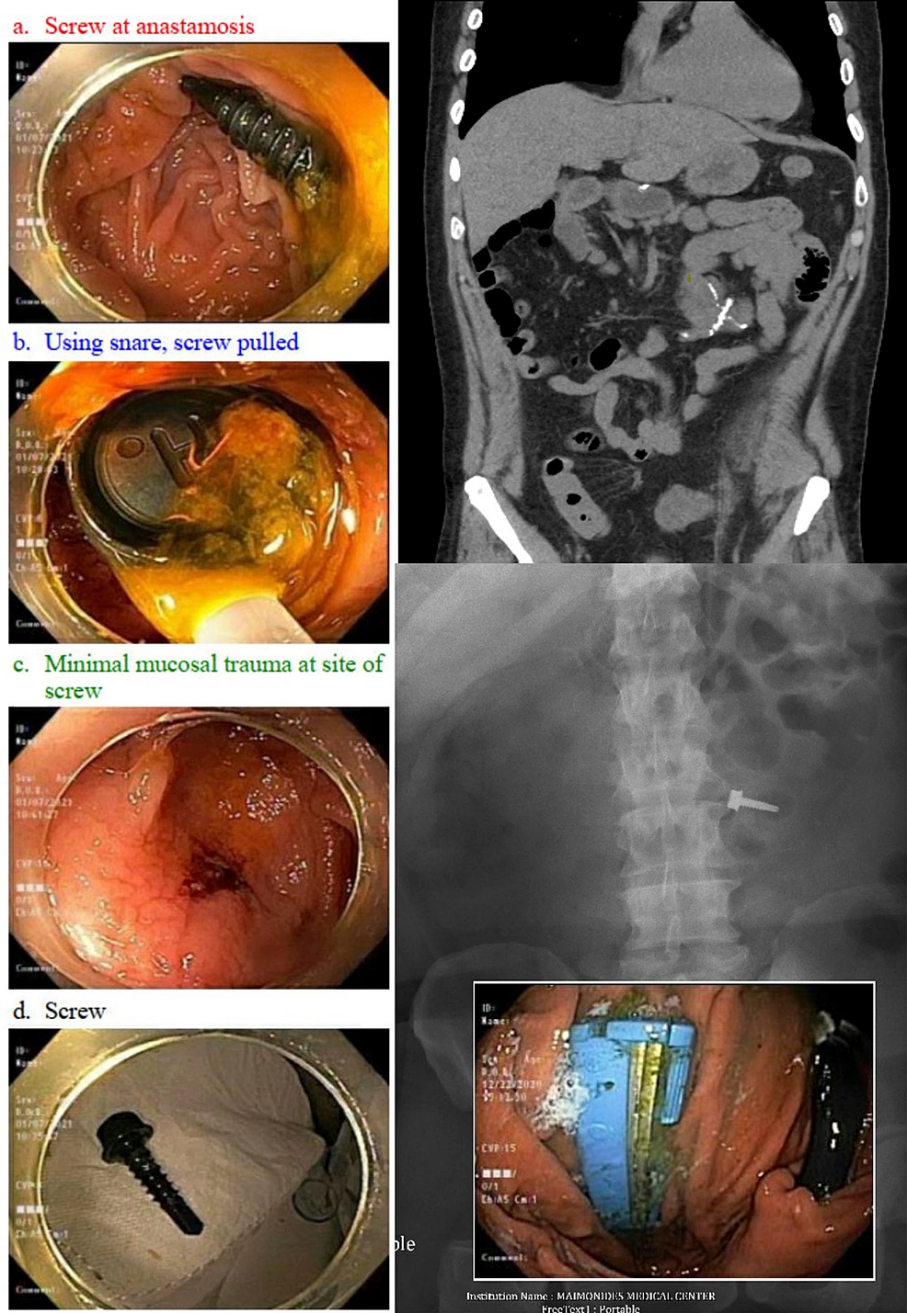
(Left panels) Colonoscopy and removal of foreign body, (top right) CT showing the previous anastomosis, (middle right) KUB showing the screw, (middle left) razor in fundus on EGD. KUB: radiograph of the kidneys, ureters, and bladder, EGD: esophagogastroduodenoscopy.

A 53-year-old male presented with an intentional ingestion of two screws, two thumbtacks, and one razor head. He has a history of opioid abuse, borderline personality disorder, depression, and recurrent foreign body ingestion, requiring three exploratory laparotomies and small bowel resection in the past. Three hours after ingestion of foreign bodies, he experienced abdominal pain and nausea. Oral mucosal examination showed one laceration. On respiratory system examination, both the hemithorax were moving equally, no tachypnea, bilateral air entry was present, no adventitious sounds were audible. Abdominal examination showed a soft, non-distended abdomen with normoactive bowel sounds, no rebound tenderness, guarding, or rigidity. Comprehensive metabolic panel and complete blood count were unremarkable. The abdomen's plain film radiographs showed metallic densities projecting at the abdomen, without any evidence of bowel obstruction or air under the diaphragm. Computed tomography (CT) scan of abdomen and pelvis without contrast showed small metallic foreign bodies in gastric antrum/pylorus, small bowel loop in left mid-abdomen, and distal descending colon (Figure 1, top right). The patient underwent emergent endoscopy for removal of gastric foreign bodies. A 2 cm shaving razor head was found in the body of the stomach, which was removed using rat tooth forceps and an endoscopy hood (Figure 1, middle left). Second look endoscopy revealed no damage to the gastric and esophageal mucosa.

As screws and thumbtacks were distal to the ligament of trietz, the decision was made to manage it conservatively as per ASGE guidelines. The patient received serial bowel regimens followed by abdominal examination and daily abdominal radiographs. He passed two thumbtacks and a screw on day 7 during his hospital stay. The subsequent imaging showed no interval migration of the last screw from ileocolic anastomosis by day 14 (Figure 1, middle right), and he was given a bowel preparation for colonoscopy. On colonoscopy, a screw was found at the ileocolic anastomosis. It was removed with a 15 mm polypectomy snare (Figure 1, left panels). Second look colonoscopy showed minimal erosion at the site of anastomosis from the tip of the screw. The patient underwent repeat computed tomography of the abdomen without contrast, negative for other foreign bodies. The patient was observed for 24 hours and discharged without any adverse events.

## Discussion

Intentional ingestion of foreign bodies is a challenging situation. In a retrospective case series by Palta et al., among 262 cases, 92% were intentional, 85% involved psychiatric patients, and 84% patients had a history of prior ingestions [1]. A variety of ingested sharp-pointed objects have been associated with complications. That is why sharp-pointed objects lodged in the esophagus are a medical emergency. Even in the absence of radiographic evidence, an endoscopic study is still advised and should be performed on an urgent or emergent basis [6]. In a retrospective analysis of 542 cases with foreign body ingestion, 75.6% of foreign bodies passed successfully, 19.5% required endoscopic removal, and 4.8% needed surgery [8]. Other studies by Ambe et al. have reproduced similar results [8]. In a PubMed-based literature review, in 80% of cases, the ingested material passed uneventfully through the gastrointestinal tract; endoscopy is performed in about 20% of cases, and surgery in less than 1% [9].

In our case, the sharp foreign body was impacted by the ileocecal valve. Although most sharp-pointed objects in the stomach will pass without incident, the risk of a complication caused by a sharp-pointed object is as high as 35% [8]. Therefore, once sharp-pointed things have migrated distally to the ligament of Treitz, daily radiographs should be done to document their passage. In our case, the gastric razor head was removed by emergent endoscopy, and foreign bodies distal to pylorus were followed with serial abdominal radiographs for progression through the gastrointestinal tract. Patients should be instructed to immediately report any change in quality or quantity of abdominal pain, vomiting, persistent temperature elevations, hematemesis, or melena. Surgical intervention should be considered for objects that fail to progress on serial radiographs after three to five days [9]. Potential indications for surgical intervention include failure of a sharp-pointed thing to progress through the intestinal tract after several days of observation, evidence of perforation, inability to remove the object endoscopically, and development of other complications such as pain, fever, bleeding, and obstruction [10].

The guidelines for endoscopic and surgical interventions for foreign body ingestion are well established. However, there are limited data to substantiate the role of colonoscopy in distally migrated foreign bodies to the colon. In our case, the non-surgical approach was chosen as the patient was at high risk for surgery given a history of three exploratory laparotomies and small bowel resection. The literature review shows a series of case reports for colonoscopy-directed management of foreign bodies distal to the ligament of Treitz, such as colonoscopic removal of two nails and a bolt from colonic flexure [11], removal of the needle from the caecum, and the Roth-net retriever-assisted withdrawal of broken glass from ascending colon [12]. More invasive extractions are exemplified by a toothpick impacting the rectosigmoid wall, removed by polypectomy snare [13]; a chicken bone perforating sigmoid mucosa, detached by endoscopic snare under laparoscopic evaluation; and a mucosal bridge entrapping a plastic twist-tie, abolished by injection sclerotherapy, electrocautery, and rat-tooth forceps [14]. In our case, a colonoscopy-directed 15 mm polypectomy snare was used to remove the screw impacted at the ileocolonic anastomosis. The screw was firmly grasped and covered with forceps to avoid mucosal injury.

As per the current ASGE guidelines, sharp objects distal to the ligament of Treitz are managed conservatively using serial abdominal examinations, bowel regimen, and serial stool monitoring for passage of the foreign body. It also recommends clinical monitoring for the development of bowel obstruction or perforation. Surgery is indicated for failed passage of foreign bodies in three to five days - no specific ASGE guidelines for colonoscopic removal of foreign bodies, which fail conservative management. By extrapolating the data from the literature review and based on our case, we recommend using an early colonoscopy as a safe and effective method for removing approachable sharp objects to reduce the risk of perforation and the need for exploratory surgery.

## Conclusions

In our case, the sharp foreign body was impacted by the ileocolonic anastomosis. Although most sharp-pointed objects in the stomach will pass without incident, the risk of a complication caused by a sharp-pointed object is as high as 35%. Once sharp-pointed things have migrated distally to the ligament of Treitz, daily radiographs should be done to document their passage. Serial abdominal examination and surgical consultation are needed. Although the failure of a sharp object to pass through the ileocecal valve is an indication for surgery, endoscopic management can be tried first.
